# Supplementary material for: An iterative gene‐editing strategy broadens eIF4E1 genetic diversity in Solanum lycopersicum and generates resistance to multiple potyvirus isolates
Source: Plant Biotechnol J. 2023 Jan 30;21(5):918–30. doi: 10.1111/pbi.14003 (PMC10106848; doi:10.1111/pbi.14003)
Supplement: Supplementary file 1 — Figure S1 Alignment of the amino acid sequences from potyvirus susceptible and resistant tomato and pepper accessions. Figure S2 Detection of insertion of the DNA donor template after the biolistic experiment with TALEN. Figure S3 Details on the target deletion using CRISPR‐Cas9 to produce eIF4E1P69T‐expressing plants after the TALEN method. Figure S4 The targets, constructs and scheme for the co‐base editing of SlALS1 and SleIF4E1 genes. Figure S5 Genomic and cDNA sequencing of P69T/M109I + G110K + A111T indicate the abnormal splicing of the eIF4E1 gene. Figure S6 eIF4E editing does not affect the eIF4E2 mRNA expression and eIF4E2 protein accumulation level in planta. Figure S7 Three‐dimensional homology modelling of the tomato eIF4E1 protein, based on crystallography data from the Pisum sativum eIF4E 3D structure (PDB ID: 2WMC‐C). [file PBI-21-918-s001.pptx]

## Slide 1
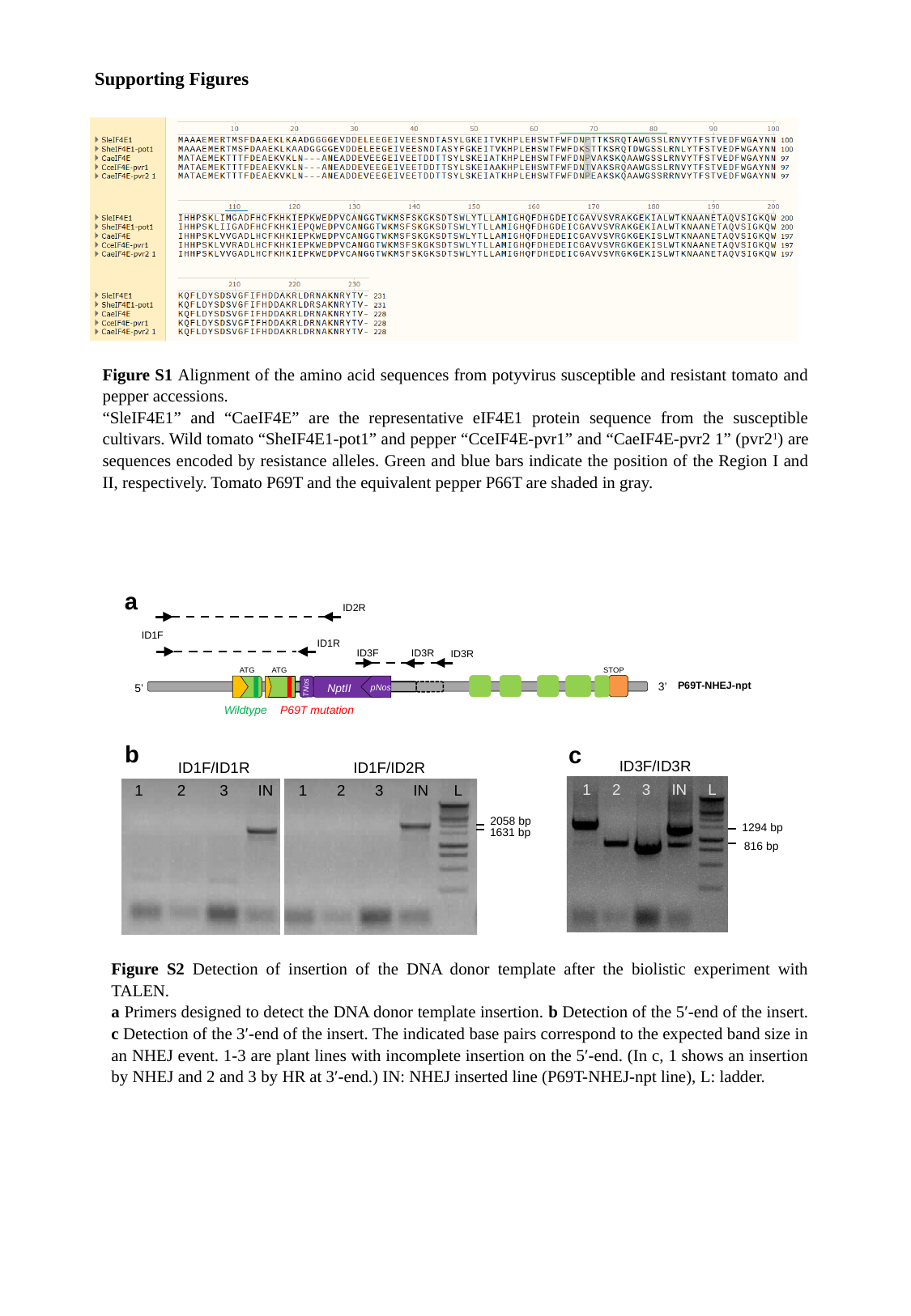

Supporting Figures
Figure S1 Alignment of the amino acid sequences from potyvirus susceptible and resistant tomato and pepper accessions.
“SleIF4E1” and “CaeIF4E” are the representative eIF4E1 protein sequence from the susceptible cultivars. Wild tomato “SheIF4E1-pot1” and pepper “CceIF4E-pvr1” and “CaeIF4E-pvr2 1” (pvr21) are sequences encoded by resistance alleles. Green and blue bars indicate the position of the Region I and II, respectively. Tomato P69T and the equivalent pepper P66T are shaded in gray.
a
ID2R
ID1F
ID1R
ID3F
ID3R
ID3R
ATG
ATG
STOP
P69T-NHEJ-npt
3’
5’
NptII
pNos
TNos
Wildtype
P69T mutation
b
ID1F/ID1R
ID1F/ID2R
 1 2 3 IN 1 2 3 IN L
2058 bp
1631 bp
c
ID3F/ID3R
1 2 3 IN L
1294 bp
816 bp
Figure S2 Detection of insertion of the DNA donor template after the biolistic experiment with TALEN.
a Primers designed to detect the DNA donor template insertion. b Detection of the 5′-end of the insert. c Detection of the 3′-end of the insert. The indicated base pairs correspond to the expected band size in an NHEJ event. 1-3 are plant lines with incomplete insertion on the 5′-end. (In c, 1 shows an insertion by NHEJ and 2 and 3 by HR at 3′-end.) IN: NHEJ inserted line (P69T-NHEJ-npt line), L: ladder.

## Slide 2
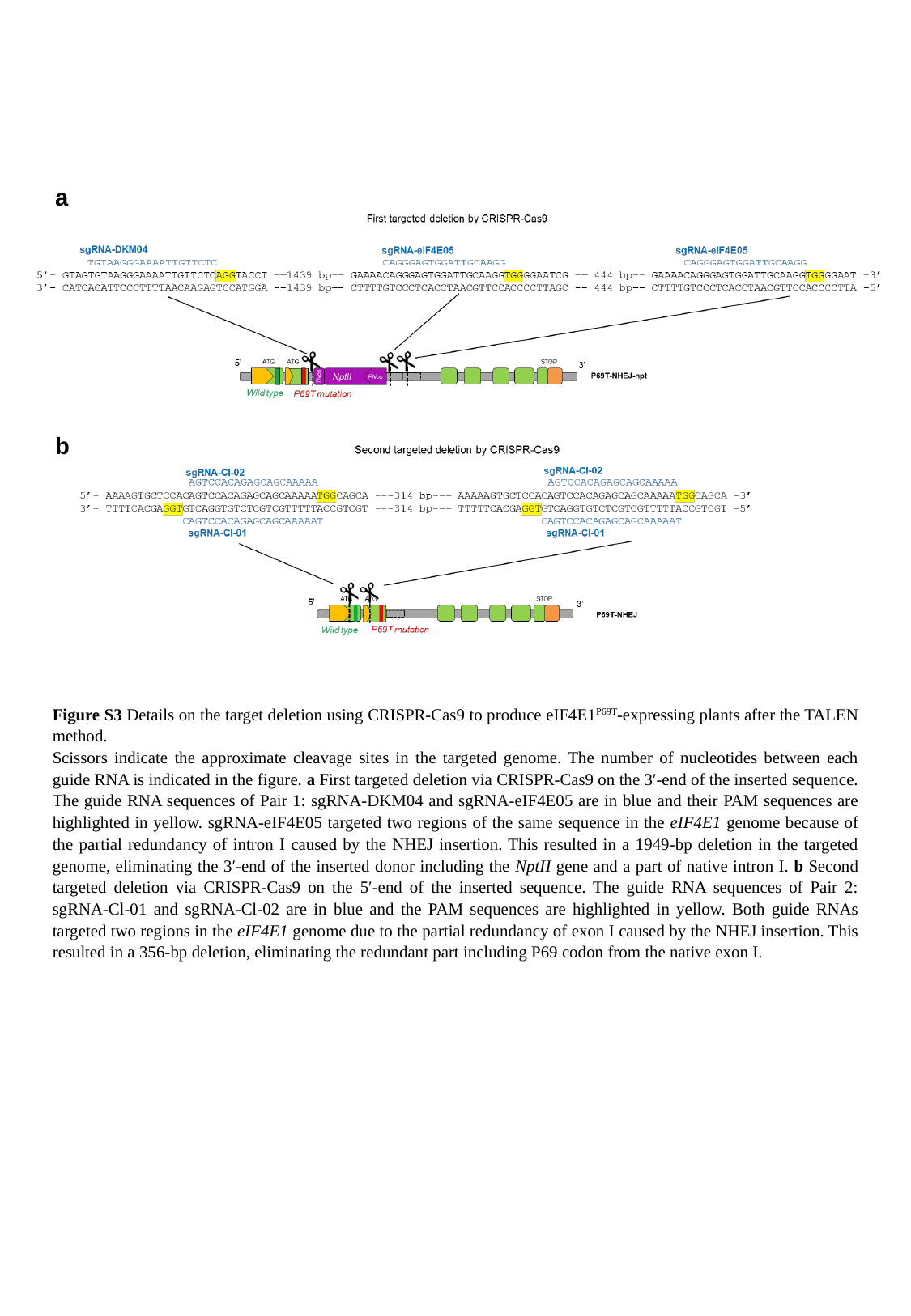

a
b
Figure S3 Details on the target deletion using CRISPR-Cas9 to produce eIF4E1P69T-expressing plants after the TALEN method.
Scissors indicate the approximate cleavage sites in the targeted genome. The number of nucleotides between each guide RNA is indicated in the figure. a First targeted deletion via CRISPR-Cas9 on the 3′-end of the inserted sequence. The guide RNA sequences of Pair 1: sgRNA-DKM04 and sgRNA-eIF4E05 are in blue and their PAM sequences are highlighted in yellow. sgRNA-eIF4E05 targeted two regions of the same sequence in the eIF4E1 genome because of the partial redundancy of intron I caused by the NHEJ insertion. This resulted in a 1949-bp deletion in the targeted genome, eliminating the 3′-end of the inserted donor including the NptII gene and a part of native intron I. b Second targeted deletion via CRISPR-Cas9 on the 5′-end of the inserted sequence. The guide RNA sequences of Pair 2: sgRNA-Cl-01 and sgRNA-Cl-02 are in blue and the PAM sequences are highlighted in yellow. Both guide RNAs targeted two regions in the eIF4E1 genome due to the partial redundancy of exon I caused by the NHEJ insertion. This resulted in a 356-bp deletion, eliminating the redundant part including P69 codon from the native exon I.

## Slide 3
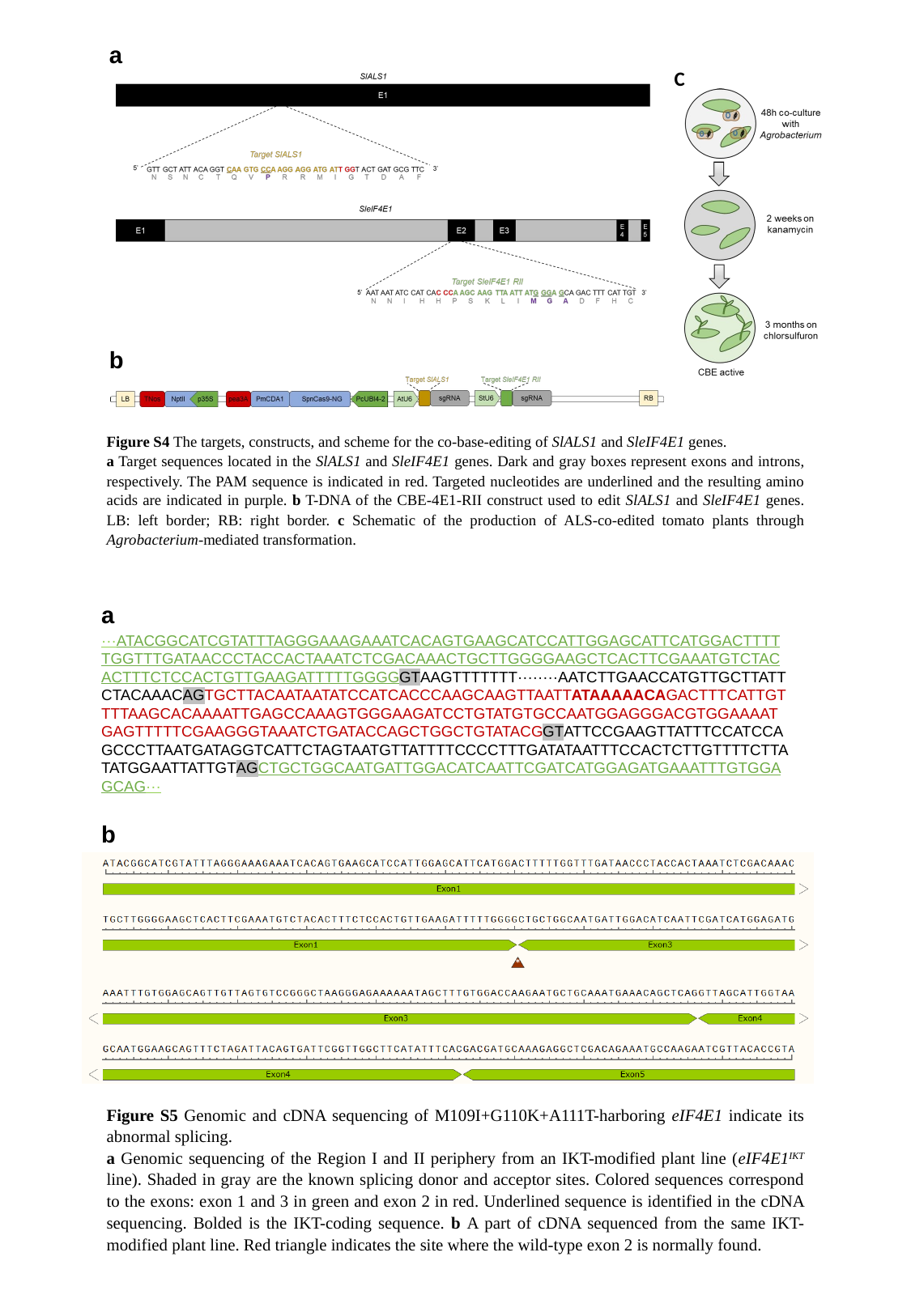

a
C
b
Figure S4 The targets, constructs, and scheme for the co-base-editing of SlALS1 and SleIF4E1 genes.
a Target sequences located in the SlALS1 and SleIF4E1 genes. Dark and gray boxes represent exons and introns, respectively. The PAM sequence is indicated in red. Targeted nucleotides are underlined and the resulting amino acids are indicated in purple. b T-DNA of the CBE-4E1-RII construct used to edit SlALS1 and SleIF4E1 genes. LB: left border; RB: right border. c Schematic of the production of ALS-co-edited tomato plants through Agrobacterium-mediated transformation.
a
···ATACGGCATCGTATTTAGGGAAAGAAATCACAGTGAAGCATCCATTGGAGCATTCATGGACTTTTTGGTTTGATAACCCTACCACTAAATCTCGACAAACTGCTTGGGGAAGCTCACTTCGAAATGTCTACACTTTCTCCACTGTTGAAGATTTTTGGGGGTAAGTTTTTTT········AATCTTGAACCATGTTGCTTATTCTACAAACAGTGCTTACAATAATATCCATCACCCAAGCAAGTTAATTATAAAAACAGACTTTCATTGTTTTAAGCACAAAATTGAGCCAAAGTGGGAAGATCCTGTATGTGCCAATGGAGGGACGTGGAAAATGAGTTTTTCGAAGGGTAAATCTGATACCAGCTGGCTGTATACGGTATTCCGAAGTTATTTCCATCCAGCCCTTAATGATAGGTCATTCTAGTAATGTTATTTTCCCCTTTGATATAATTTCCACTCTTGTTTTCTTATATGGAATTATTGTAGCTGCTGGCAATGATTGGACATCAATTCGATCATGGAGATGAAATTTGTGGAGCAG···
b
Figure S5 Genomic and cDNA sequencing of M109I+G110K+A111T-harboring eIF4E1 indicate its abnormal splicing.
a Genomic sequencing of the Region I and II periphery from an IKT-modified plant line (eIF4E1IKT line). Shaded in gray are the known splicing donor and acceptor sites. Colored sequences correspond to the exons: exon 1 and 3 in green and exon 2 in red. Underlined sequence is identified in the cDNA sequencing. Bolded is the IKT-coding sequence. b A part of cDNA sequenced from the same IKT-modified plant line. Red triangle indicates the site where the wild-type exon 2 is normally found.

## Slide 4
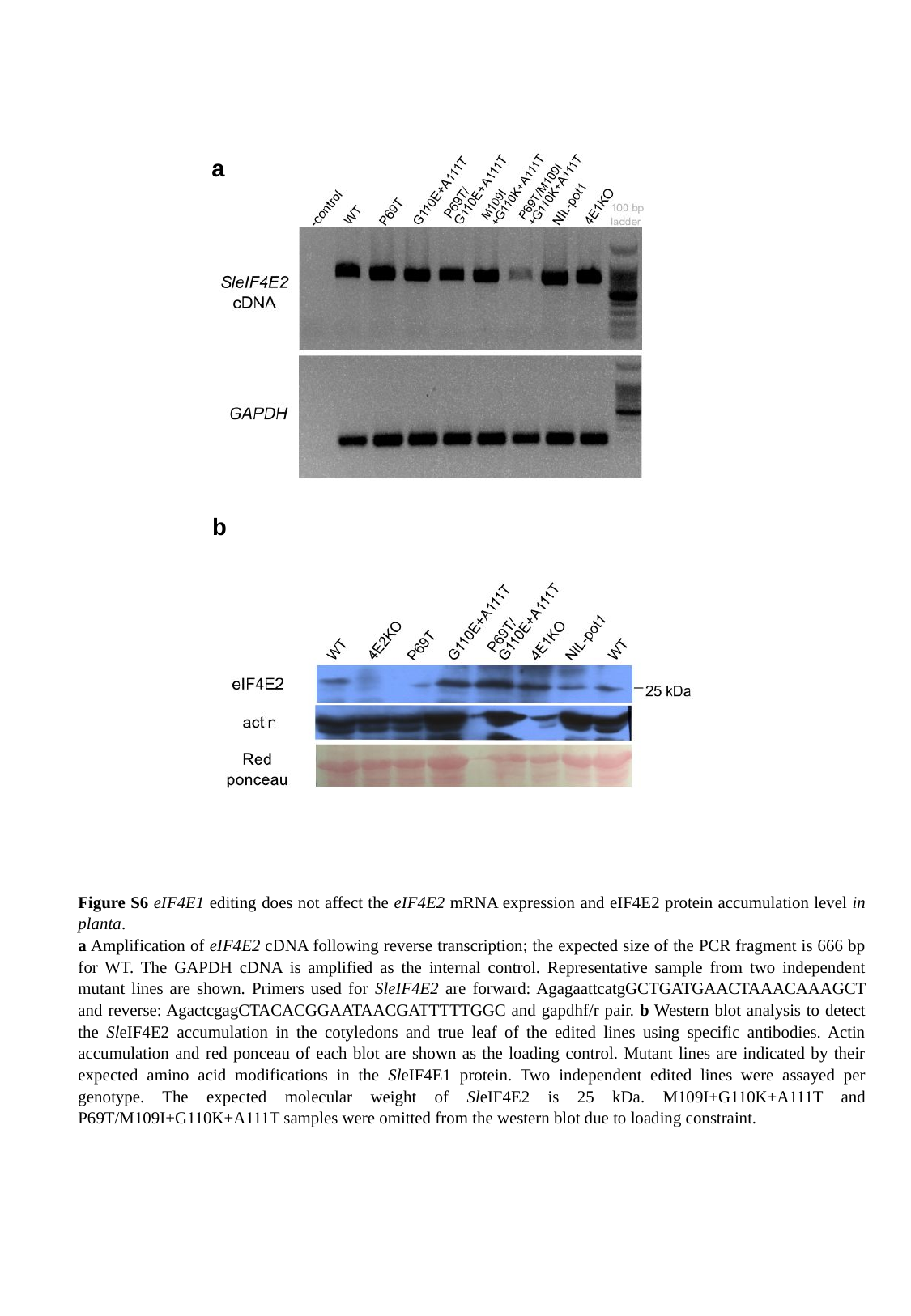

a
b
Figure S6 eIF4E1 editing does not affect the eIF4E2 mRNA expression and eIF4E2 protein accumulation level in planta.
a Amplification of eIF4E2 cDNA following reverse transcription; the expected size of the PCR fragment is 666 bp for WT. The GAPDH cDNA is amplified as the internal control. Representative sample from two independent mutant lines are shown. Primers used for SleIF4E2 are forward: AgagaattcatgGCTGATGAACTAAACAAAGCT and reverse: AgactcgagCTACACGGAATAACGATTTTTGGC and gapdhf/r pair. b Western blot analysis to detect the SleIF4E2 accumulation in the cotyledons and true leaf of the edited lines using specific antibodies. Actin accumulation and red ponceau of each blot are shown as the loading control. Mutant lines are indicated by their expected amino acid modifications in the SleIF4E1 protein. Two independent edited lines were assayed per genotype. The expected molecular weight of SleIF4E2 is 25 kDa. M109I+G110K+A111T and P69T/M109I+G110K+A111T samples were omitted from the western blot due to loading constraint.

## Slide 5
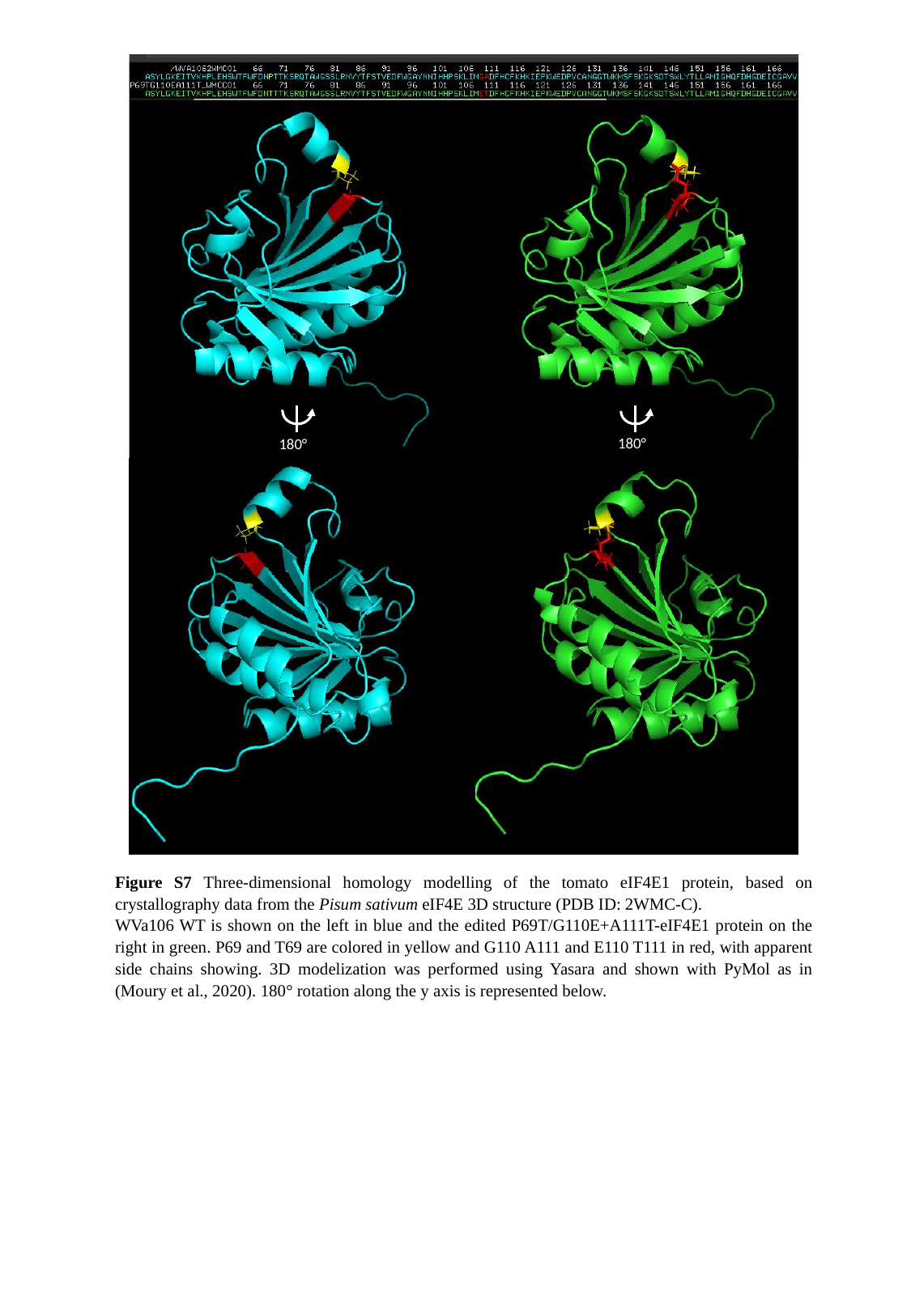

180°
180°
Figure S7 Three-dimensional homology modelling of the tomato eIF4E1 protein, based on crystallography data from the Pisum sativum eIF4E 3D structure (PDB ID: 2WMC-C).
WVa106 WT is shown on the left in blue and the edited P69T/G110E+A111T-eIF4E1 protein on the right in green. P69 and T69 are colored in yellow and G110 A111 and E110 T111 in red, with apparent side chains showing. 3D modelization was performed using Yasara and shown with PyMol as in (Moury et al., 2020). 180° rotation along the y axis is represented below.
