# Supplementary material for: An iterative gene‐editing strategy broadens eIF4E1 genetic diversity in Solanum lycopersicum and generates resistance to multiple potyvirus isolates
Source: Plant Biotechnol J. 2023 Jan 30;21(5):918–30. doi: 10.1111/pbi.14003 (PMC10106848; doi:10.1111/pbi.14003)
Supplement: Supplementary file 2 — Data S1 Sequence analysis of plant #266 with insertion of the donor template. Data S2 SleIF4E1 genomic sequence and TALEN® target sequences pCLS24222 and 24224. Data S3 The left and the right TALEN® cassette sequences in pCLS24222 and pCLS24224, respectively. Data S4 DNA donor template with P69T mutation. Data S5 The single guide RNA cassette sequence for targeting SleIF4E1. [file PBI-21-918-s003.docx]

**Supporting Information**

Supporting Data

**Data S1** Sequence analysis of plant #266 with insertion of the donor template

GCACTTCAAACAGGAGCAACTTAAGTTTGATAGAAGCTTTTCTTGTATTTTTTTTTTTGGTTAATTACTATCCTAAAGTTTTGTAATATTGAAATGTTTGCATTACTCATCTCTCATCTATCCTAGCCTCACTGAACAAATTTACATGATACATGTTGCTCCCGCGGTGATAGATATCCTTTAGAATTAGTTGAGATTTGCGAAAACTGAACTTGTGCGAAATGTTCGCATTATTCTCTTCTCATCTCTCATCAGTCCTAGCCTCGCTGAATAAATTTACATGACATATGTTGCTAGTGCTGGAGGTGATAGATATACTTTAAAATTAGTTGAAATTTGAGAAAACTGAACTTGTTCGAAATGTTTGCATTGTTCTGTTCTCATCTCTCATCTATTATAGCTTCGCAGAGTTTACCTAATAGTTATTACACCACTCTATGTGAAATGTTTGCTTTATTTTCTTCTCATCTCTCATTTGTCATAGTCTCGGTAAACAGAGTTATCTGATACCTGTTATTAATGAGAGGTGATAGAAATCCTTTAAAATTAATTGAGATGTGTGAAAACTGACCGAAACACCAACATTATAAATATAAAATATTTATAACACATGAAATCTCATTTAAATATTTATTTGAATTTCTTTCTTATTATAAAAATCACTTTTTTACTTATAAATAAAAGAAGTTCAAACTATATGATTAGCCTTACATAATTAACCAAGAAACTAAAAATTTGTAATTACATTATGACAAACAAACAAATTAATATAACTGTTCATGTGACATTATTGGTATCAAACTTCTTAATTGCTAATTTTGAAAAATAAAGCTTCCATATTCTGAACTAGATTAAAATTAAAATCCACGTAAATTAATTATTTGATTCTCACGATACCTAATTTAGGTATTTACAACATTAGTACTTCTACAATTTAAGTATTACACTATGGTCCAAACAGTTCTTATAAATACCATTTTTCTTAATTAAGTAAAAAAAAAGTGCTCCA**CAGTCCACAGAGCAGCAAAAAT**GGCAGCAGCTGAAATGGAGAGAACGATGTCGTTTGATGCAGCTGAGAAGTTGAAGGCCGCCGATGGAGGAGGAGGAGAGGTAGACGATGAACTTGAAGAAGGTGAAATTGTTGAAGAATCAAATGATACGGCATCGTATTTAGGGAAAGAAATCACAGTGAAGCATCCATTGGAGCATTCATGGACTTTTTGGTTTGATAACCCTACCACTAAATCTCGACAAACTGCTTGGGGAAGCTCACTTCGAAATGTCTACACTTTCTCCACTGTTGAAGATTTTTGGGGGTAAGTTTTTTTTTTTCCTCTTTCAAATTG**[**GTAAAAAAAAAGTGCT**CCACAGTCCACAGAGCAGCAAAAATGG**CAGCAGCTGAAATGGAGAGAACGATGTCGTTTGATGCAGCTGAGAAGTTGAAGGCCGCCGATGGAGGAGGAGGAGAGGTAGACGATGAACTTGAAGAAGGTGAAATTGTTGAAGAATCAAATGATACGGCATCGTATTTAGGGAAAGAAATCACAGTGAAGCATCCATTGGAGCATTCATGGACTTTTTGGTTTGATAACACAACCACTAAATCTCGACAAACTGCTTGGGGAAGCTCACTTCGAAATGTCTACACTTTCTCCACTGTTGAAGATTTTTGGGGGTAAGTTTTTTTTTTTCCTCTTTCAAATTGGTGATAGTGTAG**TGTAAGGGAAAAttgttctcagg**tacctcagccagacagcttgcatgccggtcgatctagtaacatagatgacaccgcgcgcgataatttatcctagtttgcgcgctatattttgttttctatcgcgtattaaatgtataattgcgggactctaatcataaaaacccatctcataaataacgtcatgcattacatgttaattattacatgcttaacgtaattcaacagaaattatatgataatcatcgcaagaccggcaacaggattcaatcttaagaaactttattgccaaatgtttgaacgatctgcttgactctagctagagtccgaaccccagagtcccgctcagaagaactcgtcaagaaggcgatagaaggcgatgcgctgcgaatcgggagcggcgataccgtaaagcacgaggaagcggtcagcccattcgccgccaagctcttcagcaatatcacgggtagccaacgctatgtcctgatagcggtccgccacacccagccggccacagtcgatgaatccagaaaagcggccattttccaccatgatattcggcaagcaggcatcgccgtgggtcacgacgagatcctcgccgtcgggcatccgcgccttgagcctggcgaacagttcggctggcgcgagcccctgatgctcttcgtccagatcatcctgatcgacaagaccggcttccatccgagtacgtgctcgctcgatgcgatgtttcgcttggtggtcgaatgggcaggtagccggatcaagcgtatgcagccgccgcattgcatcagccatgatggatactttctcggcaggagcaaggtgagatgacaggagatcctgccccggcacttcgcccaatagcagccagtcccttcccgcttcagtgacaacgtcgagcacagctgcgcaaggaacgcccgtcgtggccagccacgatagccgcgctgcctcgtcttggagttcattcagggcaccggacaggtcggtcttgacaaaaagaaccgggcgcccctgcgctgacagccggaacacggcggcatcagagcagccgattgtctgttgtgcccagtcatagccgaatagcctctccacccaagcggccggagaacctgcgtgcaatccatcttgttcaatcatgcctcgatcgagttgagagtgaatatgagactctaattggataccgaggggaatttatggaacgtcagtggagcatttttgacaagaaatatttgctagctgatagtgaccttaggcgacttttgaacgcgcaataatggtttctgacgtatgtgcttagctcattaaactccagaaacccgcggctgagtggctccttcaacgttgcggttctgtcagttccaaacgtaaaacggcttgtcccgcgtcatcggcgggggtcataacgtgactcccttaattctcatgtatgattgttctcaggtacctcagccagaTAGTGTAAGGGAAAA**CAGGGAGTGGATTGCAAGGTGG**GGAATCGAATCCTCGAGAAAGTTTAGATAGTCAATTAATTGAGCTACTGAGATTCCTCGGATTTTTTAAAAAAAATAAATTGGGGATAGAGGAAGAGGAAATGGGGGAGACTTTTATAAGGTTATACCGTTGTTAACAAGGTGAAATTTTAGGTAGTTAATCAATTGAGCTACTAAGATTCTTTAGATACGATTTTCGTAAGCTCAAATTGGAAAATAAAAAATTAGATTATTACTAAGGAGCTGAAGAAATTCAGAAATGAGTTACCTTTTGAGCCAGCAGAACCAGTTAAGTTGAGATTTCATTGATTTTAGCTTGTTTTTAATGTTGGTTGTGAATTATGGGGTTTTATTATAATGTTCCAACAATTTTGTTGGTGTTGTAGGACTTTTTTGATTTAGCGTTTTGAATGTGTTCTGATTATCTGCCTTCACATAGGTG**]**TAGTGTAAGGGAAAA**CAGGGAGTGGATTGCAAGGTGG**GGAATCGAATCCTCGAGAAAGTTTAGATAGTCAATTAATTGAGCTACTGAGATTCCTCGGATTTTTTAAAAAAAATAAATTGGGGATAGAGGAAGAGGAAATGGGGGAGACTTTTATAAGGTTATACCGTTGTTAACAAGGTGAAATTTTAGGTAGTTAATCAATTGAGCTACTAAGATTCTTTAGATACGATTTTCGTAAGCTCAAATTGGAAAATAAAAAATTAGATTATTACTAAGGAGCTGAAGAAATTCAGAAATGAGTTACCTTTTGAGCCAGCAGAACCAGTTAAGTTGAGATTTCATTGATTTTAGCTTGTTTTTAATGTTGGTTGTGAATTATGGGGTTTTATTATAATGTTCCAACAATTTTGTTGGTGTTGTAGGACTTTTTTGATTTAGCGTTTTGAATGTGTTCTGATTATCTGCCTTCACATAGGTGATATTGAGGTAAAAATGGCATCTTGATTGATTTTGCTCTCATAATTGTTAGATTTTCTTCTTGTACATTTTGCAGTTTTTAGTTAACTTGCCAAGGTGTTTAGTGGATGCACAATATGCGGTATTACTACGGAATGTCTGAATTGGTTTGAGCTTTAGTTTACCCAAGCTGTATTAGTAATTGCACATTGCTTTGTCCTTTTTTAGTTAGTACTTTCCTTAAAACTGGAATTTTGATCTCTTGGTTACTTGCCAATTTAAATTTTAGTTCATGGATCGGATTCAATGAATAGATATATGCTCTTTGGTGGTGGGAGGACAAGGATAGATAAATTAAACTCGGTAAGGAGAGAAAGTGCAGAATGAATGGAAGTTGAGACATTTTGGAGTATGGGGTGAAGAATGACCTGTGAAGTTTTAGGAAAGAAAAAGTTAACCTTGTTGAATAGTCATATTTCTCTTCCTTGCACAATCTTGTGTAGGAATGCCTTTAATTGGGTTTATATCGTCGGATAGTTTATATTGCTTCAAATCTAAGAAACTCTTTCATATTTGTCAAATGCTCCTATTTTCAACAAAGGATATTTTGAGCTGAAAAGGTAGTTGAATAATGCATTGGTCGTAGTTTTCTCTCCTAAGCAAGATAATATAGTTACTATTTTCAAGTACAACTACTTCTTTTTCTTTAGTTTACGTATAAGAGCAGGTC

*SleIF4E1* gene is in uppercase. In the pink bracket is the inserted donor sequence including 5′-UTR shaded in light yellow, exon1 in green, intron 1 in light gray, and kanamycin resistance marker colored in purple and lowercase. The P69T codon is highlighted in red. Outside of the bracket is the endogenous *SleIF4E1* sequence (native exon1 shaded in blue, 5′-UTR and intron1 not shaded).

The TALEN target region with left (red) and right (blue) binding domains are underlined. The CRISPR-gRNA targets for the deletion following the NHEJ are bolded and PAM sites are highlighted in yellow.

**Data S2** *SleIF4E*1 genomic sequence and TALEN^®^ target sequences pCLS24222 and 24224

AGTGCTCCACAGTCCACAGAGCAGCAAAAATGGCAGCAGCTGAAATGGAGAGAACGATGTCGTTTGATGCAGCTGAGAAGTTGAAGGCCGCCGATGGAGGAGGAGGAGAGGTAGACGATGAACTTGAAGAAGGTGAAATTGTTGAAGAATCAAATGATACGGCATCGTATTTAGGGAAAGAAATCACAGTGAAGCATCCATTGGAGCATTCATGGACTTTTTGGTTTGATAACCCTACCACTAAATCTCGACAAACTGCTTGGGGAAGCTCACTTCGAAATGTCTACACTTTCTCCACTGTTGAAGATTTTTGGGGGTAAGTTTTTTTT**TTTCCTCTTTCAAATTGGTGATAGTGTAGTGTAAGGGAAAACAGGGA**GTGGATTGCAAGGTGGGGAATCGAATCCTCGAGAAAGTTTAGATAGTCAATTAATTGAGCTACTGAGATTCCTCGGATTTTTTAAAAAAAATAAATTGGGGATAGAGGAAGAGGAAATGGGGGAGACTTTTATAAGGTTATACCGTTGTTAACAAGGTGAAATTTTAGGTAGTTAATCAATTGAGCTACTAAGATTCTTTAGATACGATTTTCGTAAGCTCAAATTGGAAAATAAAAAATTAGATTATTACTAAGGAGCTGAAGAAATTCAGAAATGAGTTACCTTTTGAGCCAGCAGAACCAGTTAAGTTGAGATTTCATTGATTTTAGCTTGTTTTTAATGTTGGTTGTGAATTATGGGGTTTTATTATAATGTTCCAACAATTTTGTTGGTGTTGTAGGACTTTTTTGATTTAGCGTTTTGAATGTGTTCTGATTATCTGCCTTCACATAGGTGATATTGAGGTAAAAATGGCATCTTGATTGATTTTGCTCTCATAATTGTTAGATTTTCTTCTTGTACATTTTGCAGTTTTTAGTTAACTTGCCAAGGTGTTTAGTGGATGCACAATATGCGGTATTACTACGGAATGTCTGAATTGGTTTGAGCTTTAGTTTACCCAAGCTGTATTAGTAATTGCACATTGCTTTGTCCTTTTTTAGTTAGTACTTTCCTTAAAACTGGAATTTTGATCTCTTGGTTACTTGCCAATTTAAATTTTAGTTCATGGATCGGATTCAATGAATAGATATATGCTCTTTGGTGGTGGGAGGACAAGGATAGATAAATTAAACTCGGTAAGGAGAGAAAGTGCAGAATGAATGGAAGTTGAGACATTTTGGAGTATGGGGTGAAGAATGACCTGTGAAGTTTTAGGAAAGAAAAAGTTAACCTTGTTGAATAGTCATATTTCTCTTCCTTGCACAATCTTGTGTAGGAATGCCTTTAATTGGGTTTATATCGTCGGATAGTTTATATTGCTTCAAATCTAAGAAACTCTTTCATATTTGTCAAATGCTCCTATTTTCAACAAAGGATATTTTGAGCTGAAAAGGTAGTTGAATAATGCATTGGTCGTAGTTTTCTCTCCTAAGCAAGATAATATAGTTACTATTTTCAAGTACAACTACTTCTTTTTCTTTAGTTTACGTATAAGAGCAGGTCCTGTATCTACATAGCTGTGATATCTCTATGTGCATTTGTCTTAATATACTATTCACTTGTCTTAATATATTATTCACTTTCAAAAATAAAGACAGATGTGTTTCTATTTCTTAATTAGGCTAAAGTATGCTCATCTCCTCTGTAGATTGATGAGACTTAGCTGCAGGCCAGTTTTTTCAATCTCACGCATAGACTTTTTTATTAAGTCCTATGATTTGAGTAATGTAGGTGGTAATGGTGTGAATTTGATGTCTGCCATGGATGCATGCACCTTGTTTGGTGAGTTCTTTATTGGTCTAATTACTCAAGGCACTTGAGTTATTGTACAACTTGAACGATGACATACCTGTTTGATATTCCACATCATGGATTAGGTCTTTGAAATGCTATTATCCTTTTGGCTCATGATGAAATCTTGAACCATGTTGCTTATTCTACAAACAGTGCTTACAATAATATCCATCACCCAAGCAAGTTAATTATGGGAGCAGACTTTCATTGTTTTAAGCACAAAATTGAGCCAAAGTGGGAAGATCCTGTATGTGCCAATGGAGGGACGTGGAAAATGAGTTTTTCGAAGGGTAAATCTGATACCAGCTGGCTGTATACGGTATTCCGAAGTTATTTCCATCCAGCCCTTAATGATAGGTCATTCTAGTAATGTTATTTTCCCCTTTGATATAATTTCCACTCTTGTTTTCTTATATGGAATTATTGTAGCTGCTGGCAATGATTGGACATCAATTCGATCATGGAGATGAAATTTGTGGAGCAGTTGTTAGTGTCCGGGCTAAGGGAGAAAAAATAGCTTTGTGGACCAAGAATGCTGCAAATGAAACAGCTCAGGTAATTTGCTTTATATTTTTGGTGTCAACGGCCACGTTTGTCATTTTCTTGGATCAAGCGGACAGACATTTTTTGTTGTGTACATACTGTAGTGCTGATGTTTATTCAAAATATACTATGAAATACATTGTACCGTTGAGTATATTGGAAGTTAAGAGGGGAAGTCATCAGATATATAGGAAGTGCTCCTTGTGAGAAATTTCTCTAGTAGGGCTCAAACCCGAGAACCATGGTTAAGGACGGAGGGATCCCTTTGGTGGTATCAAGGGTTAATTATATTATTTCATGTTTCATATGATTGAAACATTTGGATATCCTTGCAAAGTGTGATATTGTTTTGTCTCGTCATTTGTTTTATGCTTCATAAGAATTATCTTTTCTTTCGTCCCCTTCTCTTTGTTTCTTTTTCTCCAGTGTACTTCATTCTTCTAGAATTATATAGTGGTCGGCGTTCCCTTTTGTGCTTCATTGATAATATTGAATCCTTTCAGCTGTTTACCGGTTGATTATTTTTATGTTGGTGCTTATTGCTAGTTTTTTGTTTTTATATCTTTGCAACTATACCTACCATCTTCCTGAAACTTCTCTTTACAGGTTAGCATTGGTAAGCAATGGAAGCAGTTTCTAGATTACAGTGATTCGGTTGGCTTCATATTTCACGTATGAAATCTTGGTTATCATACGCCTTTAATTCAGTTTCTCTTCAATTAGCAAGACTCATAAAGAATCATCTTCTTTTGCAGGACGATGCAAAGAGGCTCGACAGAAATGCCAAGAATCGTTACACCGTATAGTTCTTGATGCAGTGTGGGATTGCAAGAAACACAATTCGTACTGGAAAGGTTGGTAGGTACTAGTTTAGTTTCTCATTTGATAAGCTTCTGGTTTGAGTAACTCGTGTGTTGGTGTTTACACTTTCTAATCGTGGAAAATTGTTTGATTTGAATCCATGCCTCTATGTTTCGTGTGTTCTGTGTTACTTTCCCAGCTTCCCTATTATTTCTCTGCAGTAAAAATCTCTGAAAATATTGTTGATGGAGGTGAAAGTGATACCAAGCACATAACAAAACACAAAT

The TAL effector target region is underlined. And the left and the right TAL effector DNA-binding domains are in red and blue, respectively. The exons are shaded in blue.

**Data S3** The left and the right TALEN^®^ cassette sequences in pCLS24222 and pCLS24224, respectively

(The left TALEN^®^ cassette)

aatcccacaaaaatctgagcttaacagcacagttgctcctctcagagcagaatcgggtattcaacaccctcatatcaactactacgttgtgtataacggtccacatgccggtatatacgatgactggggttgtacaaaggcggcaacaaacggcgttcccggagttgcacacaagaaatttgccactattacagaggcaagagcagcagctgacgcgtacacaacaagtcagcaaacagacaggttgaacttcatccccaaaggagaagctcaactcaagcccaagagctttgctaaggccctaacaagcccaccaaagcaaaaagcccactggctcacgctaggaaccaaaaggcccagcagtgatccagccccaaaagagatctcctttgccccggagattacaatggacgatttcctctatctttacgatctaggaaggaagttcgaaggtgaaggtgacgacactatgttcaccactgataatgagaaggttagcctcttcaatttcagaaagaatgctgacccacagatggttagagaggcctacgcagcaggtctcatcaagacgatctacccgagtaacaatctccaggagatcaaataccttcccaagaaggttaaagatgcagtcaaaagattcaggactaattgcatcaagaacacagagaaagacatatttctcaagatcagaagtactattccagtatggacgattcaaggcttgcttcataaaccaaggcaagtaatagagattggagtctctaaaaaggtagttcctactgaatctaaggccatgcatggagtctaagattcaaatcgaggatctaacagaactcgccgtgaagactggcgaacagttcatacagagtcttttacgactcaatgacaagaagaaaatcttcgtcaacatggtggagcacgacactctggtctactccaaaaatgtcaaagatacagtctcagaagaccaaagggctattgagacttttcaacaaaggataatttcgggaaacctcctcggattccattgcccagctatctgtcacttcatcgaaaggacagtagaaaaggaaggtggctcctacaaatgccatcattgcgataaaggaaaggctatcattcaagatctctctgccgacagtggtcccaaagatggacccccacccacgaggagcatcgtggaaaaagaagacgttccaaccacgtcttcaaagcaagtggattgatgtgacatctccactgacgtaagggatgacgcacaatcccactatccttcgcaagacccttcctctatataaggaagttcatttcatttggagaggacacgcacgagatctgcaggtcgactctagaggcgcgccacacgcaaacacaaatacacagcggccttgccaccatgggcgatcctaaaaagaaacgtaaggtcatcgattacccatacgatgttccagattacgctatcgatatcgccgatctacgcacgctcggctacagccagcagcaacaggagaagatcaaaccgaaggttcgttcgacagtggcgcagcaccacgaggcactggtcggccacgggtttacacacgcgcacatcgttgcgttaagccaacacccggcagcgttagggaccgtcgctgtcaagtatcaggacatgatcgcagcgttgccagaggcgacacacgaagcgatcgttggcgtcggcaaacagtggtccggcgcacgcgctctggaggccttgctcacggtggcgggagagttgagaggtccaccgttacagttggacacaggccaacttctcaagattgcaaaacgtggcggcgtgaccgcagtggaggcagtgcatgcatggcgcaatgcactgacgggtgccccgctcaacttgaccccccagcaggtggtggccatcgccagcaatggcggtggcaagcaggcgctggagacggtccagcggctgttgccggtgctgtgccaggcccacggcttgaccccccagcaggtggtggccatcgccagcaatggcggtggcaagcaggcgctggagacggtccagcggctgttgccggtgctgtgccaggcccacggcttgaccccggagcaggtggtggccatcgccagccacgatggcggcaagcaggcgctggagacggtccagcggctgttgccggtgctgtgccaggcccacggcttgaccccggagcaggtggtggccatcgccagccacgatggcggcaagcaggcgctggagacggtccagcggctgttgccggtgctgtgccaggcccacggcttgaccccccagcaggtggtggccatcgccagcaatggcggtggcaagcaggcgctggagacggtccagcggctgttgccggtgctgtgccaggcccacggcttgaccccggagcaggtggtggccatcgccagccacgatggcggcaagcaggcgctggagacggtccagcggctgttgccggtgctgtgccaggcccacggcttgaccccccagcaggtggtggccatcgccagcaatggcggtggcaagcaggcgctggagacggtccagcggctgttgccggtgctgtgccaggcccacggcttgaccccccagcaggtggtggccatcgccagcaatggcggtggcaagcaggcgctggagacggtccagcggctgttgccggtgctgtgccaggcccacggcttgaccccccagcaggtggtggccatcgccagcaatggcggtggcaagcaggcgctggagacggtccagcggctgttgccggtgctgtgccaggcccacggcttgaccccggagcaggtggtggccatcgccagccacgatggcggcaagcaggcgctggagacggtccagcggctgttgccggtgctgtgccaggcccacggcttgaccccggagcaggtggtggccatcgccagcaatattggtggcaagcaggcgctggagacggtgcaggcgctgttgccggtgctgtgccaggcccacggcttgaccccggagcaggtggtggccatcgccagcaatattggtggcaagcaggcgctggagacggtgcaggcgctgttgccggtgctgtgccaggcccacggcttgaccccggagcaggtggtggccatcgccagcaatattggtggcaagcaggcgctggagacggtgcaggcgctgttgccggtgctgtgccaggcccacggcttgaccccccagcaggtggtggccatcgccagcaatggcggtggcaagcaggcgctggagacggtccagcggctgttgccggtgctgtgccaggcccacggcttgaccccccagcaggtggtggccatcgccagcaatggcggtggcaagcaggcgctggagacggtccagcggctgttgccggtgctgtgccaggcccacggcttgacccctcagcaggtggtggccatcgccagcaatggcggcggcaggccggcgctggagagcattgttgcccagttatctcgccctgatccggcgttggccgcgttgaccaacgaccacctcgtcgccttggcctgcctcggcgggcgtcctgcgctggatgcagtgaaaaagggattgggggatcctatcagccgttcccagctggtgaagtccgagctggaggagaagaaatccgagttgaggcacaagctgaagtacgtgccccacgagtacatcgagctgatcgagatcgcccggaacagcacccaggaccgtatcctggagatgaaggtgatggagttcttcatgaaggtgtacggctacaggggcaagcacctgggcggctccaggaagcccgacggcgccatctacaccgtgggctcccccatcgactacggcgtgatcgtggacaccaaggcctactccggcggctacaacctgcccatcggccaggccgacgaaatgcagaggtacgtggaggagaaccagaccaggaacaagcacatcaaccccaacgagtggtggaaggtgtacccctccagcgtgaccgagttcaagttcctgttcgtgtccggccacttcaagggcaactacaaggcccagctgaccaggctgaaccacatcaccaactgcaacggcgccgtgctgtccgtggaggagctcctgatcggcggcgagatgatcaaggccggcaccctgaccctggaggaggtgaggaggaagttcaacaacggcgagatcaacttcgcggccgactgataagtcgaccaccaccaccaccaccactgataagagctcctcgagcgccggcgagcctcgaatttccccgatcgttcaaacatttggcaataaagtttcttaagattgaatcctgttgccggtcttgcgatgattatcatataatttctgttgaattacgttaagcatgtaataattaacatgtaatgcatgacgttatttatgagatgggtttttatgattagagtcccgcaattatacatttaatacgcgatagaaaacaaaatatagcgcgcaaactaggataaattatcgcgcgcggtgtcatctatgttactagatcgggaattcgtaatcatggtcatagctgtttcctgtgtgaaattgttatccgctcacaattccacacaacatacgagccggaagcataaagtgtaaagcctggggtgcctaatgagtgagctaactcacattaattgcgttgcgc

(The right TALEN^®^ cassette)

aatcccacaaaaatctgagcttaacagcacagttgctcctctcagagcagaatcgggtattcaacaccctcatatcaactactacgttgtgtataacggtccacatgccggtatatacgatgactggggttgtacaaaggcggcaacaaacggcgttcccggagttgcacacaagaaatttgccactattacagaggcaagagcagcagctgacgcgtacacaacaagtcagcaaacagacaggttgaacttcatccccaaaggagaagctcaactcaagcccaagagctttgctaaggccctaacaagcccaccaaagcaaaaagcccactggctcacgctaggaaccaaaaggcccagcagtgatccagccccaaaagagatctcctttgccccggagattacaatggacgatttcctctatctttacgatctaggaaggaagttcgaaggtgaaggtgacgacactatgttcaccactgataatgagaaggttagcctcttcaatttcagaaagaatgctgacccacagatggttagagaggcctacgcagcaggtctcatcaagacgatctacccgagtaacaatctccaggagatcaaataccttcccaagaaggttaaagatgcagtcaaaagattcaggactaattgcatcaagaacacagagaaagacatatttctcaagatcagaagtactattccagtatggacgattcaaggcttgcttcataaaccaaggcaagtaatagagattggagtctctaaaaaggtagttcctactgaatctaaggccatgcatggagtctaagattcaaatcgaggatctaacagaactcgccgtgaagactggcgaacagttcatacagagtcttttacgactcaatgacaagaagaaaatcttcgtcaacatggtggagcacgacactctggtctactccaaaaatgtcaaagatacagtctcagaagaccaaagggctattgagacttttcaacaaaggataatttcgggaaacctcctcggattccattgcccagctatctgtcacttcatcgaaaggacagtagaaaaggaaggtggctcctacaaatgccatcattgcgataaaggaaaggctatcattcaagatctctctgccgacagtggtcccaaagatggacccccacccacgaggagcatcgtggaaaaagaagacgttccaaccacgtcttcaaagcaagtggattgatgtgacatctccactgacgtaagggatgacgcacaatcccactatccttcgcaagacccttcctctatataaggaagttcatttcatttggagaggacacgcacgagatctgcaggtcgactctagaggcgcgccacacgcaaacacaaatacacagcggccttgccaccatgggcgatcctaaaaagaaacgtaaggtcatcgataaggagaccgccgctgccaagttcgagagacagcacatggacagcatcgatatcgccgatctacgcacgctcggctacagccagcagcaacaggagaagatcaaaccgaaggttcgttcgacagtggcgcagcaccacgaggcactggtcggccacgggtttacacacgcgcacatcgttgcgttaagccaacacccggcagcgttagggaccgtcgctgtcaagtatcaggacatgatcgcagcgttgccagaggcgacacacgaagcgatcgttggcgtcggcaaacagtggtccggcgcacgcgctctggaggccttgctcacggtggcgggagagttgagaggtccaccgttacagttggacacaggccaacttctcaagattgcaaaacgtggcggcgtgaccgcagtggaggcagtgcatgcatggcgcaatgcactgacgggtgccccgctcaacttgaccccggagcaggtggtggccatcgccagccacgatggcggcaagcaggcgctggagacggtccagcggctgttgccggtgctgtgccaggcccacggcttgaccccggagcaggtggtggccatcgccagccacgatggcggcaagcaggcgctggagacggtccagcggctgttgccggtgctgtgccaggcccacggcttgaccccggagcaggtggtggccatcgccagccacgatggcggcaagcaggcgctggagacggtccagcggctgttgccggtgctgtgccaggcccacggcttgaccccccagcaggtggtggccatcgccagcaatggcggtggcaagcaggcgctggagacggtccagcggctgttgccggtgctgtgccaggcccacggcttgaccccccagcaggtggtggccatcgccagcaataatggtggcaagcaggcgctggagacggtccagcggctgttgccggtgctgtgccaggcccacggcttgaccccccagcaggtggtggccatcgccagcaatggcggtggcaagcaggcgctggagacggtccagcggctgttgccggtgctgtgccaggcccacggcttgaccccccagcaggtggtggccatcgccagcaatggcggtggcaagcaggcgctggagacggtccagcggctgttgccggtgctgtgccaggcccacggcttgaccccccagcaggtggtggccatcgccagcaatggcggtggcaagcaggcgctggagacggtccagcggctgttgccggtgctgtgccaggcccacggcttgaccccccagcaggtggtggccatcgccagcaatggcggtggcaagcaggcgctggagacggtccagcggctgttgccggtgctgtgccaggcccacggcttgaccccggagcaggtggtggccatcgccagccacgatggcggcaagcaggcgctggagacggtccagcggctgttgccggtgctgtgccaggcccacggcttgaccccggagcaggtggtggccatcgccagccacgatggcggcaagcaggcgctggagacggtccagcggctgttgccggtgctgtgccaggcccacggcttgaccccggagcaggtggtggccatcgccagccacgatggcggcaagcaggcgctggagacggtccagcggctgttgccggtgctgtgccaggcccacggcttgaccccccagcaggtggtggccatcgccagcaatggcggtggcaagcaggcgctggagacggtccagcggctgttgccggtgctgtgccaggcccacggcttgaccccccagcaggtggtggccatcgccagcaatggcggtggcaagcaggcgctggagacggtccagcggctgttgccggtgctgtgccaggcccacggcttgaccccggagcaggtggtggccatcgccagcaatattggtggcaagcaggcgctggagacggtgcaggcgctgttgccggtgctgtgccaggcccacggcttgacccctcagcaggtggtggccatcgccagcaatggcggcggcaggccggcgctggagagcattgttgcccagttatctcgccctgatccggcgttggccgcgttgaccaacgaccacctcgtcgccttggcctgcctcggcgggcgtcctgcgctggatgcagtgaaaaagggattgggggatcctatcagccgttcccagctggtgaagtccgagctggaggagaagaaatccgagttgaggcacaagctgaagtacgtgccccacgagtacatcgagctgatcgagatcgcccggaacagcacccaggaccgtatcctggagatgaaggtgatggagttcttcatgaaggtgtacggctacaggggcaagcacctgggcggctccaggaagcccgacggcgccatctacaccgtgggctcccccatcgactacggcgtgatcgtggacaccaaggcctactccggcggctacaacctgcccatcggccaggccgacgaaatgcagaggtacgtggaggagaaccagaccaggaacaagcacatcaaccccaacgagtggtggaaggtgtacccctccagcgtgaccgagttcaagttcctgttcgtgtccggccacttcaagggcaactacaaggcccagctgaccaggctgaaccacatcaccaactgcaacggcgccgtgctgtccgtggaggagctcctgatcggcggcgagatgatcaaggccggcaccctgaccctggaggaggtgaggaggaagttcaacaacggcgagatcaacttcgcggccgactgataagtcgaccaccaccaccaccaccactgataagagctcctcgagcgccggcgagcctcgaatttccccgatcgttcaaacatttggcaataaagtttcttaagattgaatcctgttgccggtcttgcgatgattatcatataatttctgttgaattacgttaagcatgtaataattaacatgtaatgcatgacgttatttatgagatgggtttttatgattagagtcccgcaattatacatttaatacgcgatagaaaacaaaatatagcgcgcaaactaggataaattatcgcgcgcggtgtcatctatgttactagatcgggaattcgtaatcatggtcatagctgtttcctgtgtgaaattgttatccgctcacaattccacacaacatacgagccggaagcataaagtgtaaagcctggggtgcctaatgagtgagctaactcacattaattgcgttgcgctcactgcccgctttccagtcgggaaacctgtcgtgccagctgcattaat

Codons corresponding to the repeat Variable Diresidue (RVD), specific to A (NI) are highlighted in blue, specific to T (NG) in red, specific to G (NN) in yellow, and specific to C (HD) in green. Nucleotide sequence recognized by pCLS24222 is TTTCCTCTTTCAAATT**T** and by pCLS24224 is TCCCTGTTTTCCCTTA**T** (its reverse complement: **A**TAAGGGAAAACAGGGA. The target sequence is preceded by a **T** required for TALEN activity (Moscou and Bogdanove, 2009; Cermak et al., 2011). The pCaMV35S is in light blue, the TAL effector repeat array in black, the nonspecific DNA cleavage domain of the FokI endonuclease in orange. Nos terminator in green. The SV40 NLS is underlined.

**Data S4** DNA donor template with P69T mutation

aaataatgattttattttgactgatagtgacctgttcgttgcaacacattgatgagcaatgcttttttataatgccaactttgtacaaaaaagcagTTTTGAAAAATAAAGCTTCCATATTCTGAACTAGATTAAAATTAAAATCCACGTAAATTAATTATTTGATTCTCACGATACCTAATTTAGGTATTTACAACATTAGTACTTCTACAATTTAAGTATTACACTATGGTCCAAACAGTTCTTATAAATACCATTTTTCTTAATTAAGTAAAAAAAAAGTGCTCCACAGTCCACAGAGCAGCAAAAATGGCAGCAGCTGAAATGGAGAGAACGATGTCGTTTGATGCAGCTGAGAAGTTGAAGGCCGCCGATGGAGGAGGAGGAGAGGTAGACGATGAACTTGAAGAAGGTGAAATTGTTGAAGAATCAAATGATACGGCATCGTATTTAGGGAAAGAAATCACAGTGAAGCATCCATTGGAGCATTCATGGACTTTTTGGTTTGATAACACAACCACTAAATCTCGACAAACTGCTTGGGGAAGCTCACTTCGAAATGTCTACACTTTCTCCACTGTTGAAGATTTTTGGGGGTAAGTTTTTTTTTTTCCTCTTTCAAATTGGTGATAGTGTAGTGTAAGGGAAAAttgttctcaggtacctcagccagacagcttgcatgccggtcgatctagtaacatagatgacaccgcgcgcgataatttatcctagtttgcgcgctatattttgttttctatcgcgtattaaatgtataattgcgggactctaatcataaaaacccatctcataaataacgtcatgcattacatgttaattattacatgcttaacgtaattcaacagaaattatatgataatcatcgcaagaccggcaacaggattcaatcttaagaaactttattgccaaatgtttgaacgatctgcttgactctagctagagtccgaaccccagagtcccgctcagaagaactcgtcaagaaggcgatagaaggcgatgcgctgcgaatcgggagcggcgataccgtaaagcacgaggaagcggtcagcccattcgccgccaagctcttcagcaatatcacgggtagccaacgctatgtcctgatagcggtccgccacacccagccggccacagtcgatgaatccagaaaagcggccattttccaccatgatattcggcaagcaggcatcgccgtgggtcacgacgagatcctcgccgtcgggcatccgcgccttgagcctggcgaacagttcggctggcgcgagcccctgatgctcttcgtccagatcatcctgatcgacaagaccggcttccatccgagtacgtgctcgctcgatgcgatgtttcgcttggtggtcgaatgggcaggtagccggatcaagcgtatgcagccgccgcattgcatcagccatgatggatactttctcggcaggagcaaggtgagatgacaggagatcctgccccggcacttcgcccaatagcagccagtcccttcccgcttcagtgacaacgtcgagcacagctgcgcaaggaacgcccgtcgtggccagccacgatagccgcgctgcctcgtcttggagttcattcagggcaccggacaggtcggtcttgacaaaaagaaccgggcgcccctgcgctgacagccggaacacggcggcatcagagcagccgattgtctgttgtgcccagtcatagccgaatagcctctccacccaagcggccggagaacctgcgtgcaatccatcttgttcaatcatgcctcgatcgagttgagagtgaatatgagactctaattggataccgaggggaatttatggaacgtcagtggagcatttttgacaagaaatatttgctagctgatagtgaccttaggcgacttttgaacgcgcaataatggtttctgacgtatgtgcttagctcattaaactccagaaacccgcggctgagtggctccttcaacgttgcggttctgtcagttccaaacgtaaaacggcttgtcccgcgtcatcggcgggggtcataacgtgactcccttaattctcatgtatgattgttctcaggtacctcagccagaTAGTGTAAGGGAAAACAGGGAGTGGATTGCAAGGTGGGGAATCGAATCCTCGAGAAAGTTTAGATAGTCAATTAATTGAGCTACTGAGATTCCTCGGATTTTTTAAAAAAAATAAATTGGGGATAGAGGAAGAGGAAATGGGGGAGACTTTTATAAGGTTATACCGTTGTTAACAAGGTGAAATTTTAGGTAGTTAATCAATTGAGCTACTAAGATTCTTTAGATACGATTTTCGTAAGCTCAAATTGGAAAATAAAAAATTAGATTATTACTAAGGAGCTGAAGAAATTCAGAAATGAGTTACCTTTTGAGCCAGCAGAACCAGTTAAGTTGAGATTTCATTGATTTTAGCTTGTTTTTAATGTTGGTTGTGAATTATGGGGTTTTATTATAATGTTCCAACAATTTTGTTGGTGTTGTAGGACTTTTTTGATTTAGCGTTTTGAATGTGTTCTGATTATCTGCCTTCACATAGGTGgtttaaaccagctttcttgtacaaagttggcattataagaaagcattgcttatcaatttgttgcaacgaacaggtcactatcagtcaaaataaaatcattattt

*eIF4E1* gene is in uppercase. 5′-UTR is shaded in light yellow, exon1 in green, intron 1 in light gray. PacI and PmeI restriction sites are highlighted in pink and dark green, respectively. The P69T codon is highlighted in red.

The kanamycin resistance marker that consists of the *Nos* promoter, the *NptII* gene, and the *Nos* terminator is in purple. Two RAG1 target sequences are underlined (not used in this work).

**Data S5** The single guide RNA cassette sequence for targeting *SleIF4E1*

GGGGACAAGTTTGTACAAAAAAGCAGGCTTCACTAGTGTTCAGTTGCATTATGTCTTTATACACCCCTACCTAGATGATTAAGTTTTACTTTAGTTGGTGTGAAATGGATAAATTCTAAAATATGAGGTGTGGAATGAAGGATTGTCATCAATTAGTTGGCCCCAACCAAGTAAAATAAGAAGGCCGGCCCATTACAATTAAGTCGTCACACAAGTGGGCTTCATTGAAACAAGCGCAAAAAGGAGTCCAGGCCCGTGTTAGCGTGAAGACTCAACCAGCGATTTCTCCCTCATCGGTTGCACAGAAAAGCTGTGTGTTGTTTATATGGCGAAACCTAACAGTCTGACTTG**CTCCCATAATTAACTTGCTT**GTTTAAGAGCTATGCTGGAAACAGCATAGCAAGTTTAAATAAGGCTAGTCCGTTATCAACTTGAAAAAGTGGCACCGAGTCGGTGCTTTTTTTGTTTTTTATGTCTAGCGGAGGTAGTGAGCTCGGCTCTAGCGGGCCATTTATATGGGAGAGTGCCACCACGCGTGAAACTCCTGGCCCTGAGGACTAGTACCCAGCTTTCTTGTACAAAGTGGTCCCC

Sequence of the guide cassette cloned into the pDicAID_nCas9-PmCDA1_NptII-SlALS1 through *SpeI* restriction/ligation, resulting into the CBE-4E1-RII. Gateway AttB1/B2 sequences are colored in blue, *SpeI* restriction sites in red, *StU6* promoter sequence in yellow, 20-bp target sequence in green, sgRNA scaffold sequence in purple.
