# Supplementary material for: An iterative gene‐editing strategy broadens eIF4E1 genetic diversity in Solanum lycopersicum and generates resistance to multiple potyvirus isolates
Source: Plant Biotechnol J. 2023 Jan 30;21(5):918–30. doi: 10.1111/pbi.14003 (PMC10106848; doi:10.1111/pbi.14003)
Supplement: Supplementary file 3 — Table S1 Number of the individuals used and obtained for the Region I codon insertion. Table S2 Synthesis of all nucleases and target sites used. Table S3 List of primers used in this study. [file PBI-21-918-s002.pdf]

Supporting Tables

Table S1: Number of the individuals used and obtained for the Region I codon insertion.

Initial leaves used in biolistic	Leaf pieces cut out	Buds that regenerated on the selective media	NHEJ (both 5′ and 3′ ends) event	HR event
40	233	32	1	0

Table S2: Synthesis of all nucleases and target sites used.

Guide name	Target	Detection
sgRNA-DKM04	TGTAAGGGAAAATTGTTCTC**AGG**	Pair 1; NHEJ 3′-end deletion
sgRNA-eIF4E05	CAGGGAGTGGATTGCAAGG**TGG**	
sgRNA-Cl-01	**CCA**CAGTCCACAGAGCAGCAAAAAT	Pair 2; NHEJ 5′-end deletion
sgRNA-Cl-02	AGTCCACAGAGCAGCAAAAA**TGG**	

Table S3: List of primers used in this study.

Primer	Sequence 5′-3′	Amplicon size (bp)	Detection
ID1F	TGCGAAAACTGAACTTGTGC	1275 if HR, 1631 if NHEJ	DNA donor template insertion in *eIF4E* gene 5′ end
ID1R	CAAAATATAGCGCGCAAACTAGG		
ID1F	TGCGAAAACTGAACTTGTGC	1702 if HR, 2058 if NHEJ	DNA donor template insertion in *eIF4E* gene 3′ end
ID2R	ATGGCCGCTTTTCTGGATTC		
ID3F	GAACCTGCGTGCAATCCATC	816 if HR, 816 and 1294 if NHEJ	DNA donor template insertion in *eIF4E* gene 3′ end
ID3R	AGGCAGATAATCAGAACACATTCA		
Fok1F	AATCCGAGTTGAGGCACAAG	445	TALENs Fok1 domain
Fok1R	TTGCAGTTGGTGATGTGGTT		
Cas9F	TCCCTTACTACGTGGGACCTC	1438	*Cas9* gene
Cas9R	ATCTGCCTGGTTTCCACAAG		
DD1F	AACGATGTCGTTTGATGCAG	2267; 788 if deleted	*NptII* gene deletion (NHEJ 3′-end deletion)
DD2R	TCAAGATGCCATTTTTACCTCA		
DD3F	AACTGTTCATGTGACATTATTGGT	1340; 940 if deleted	NHEJ 5′-end deletion
DD4R	TCAAGATGCCATTTTTACCTCA		
Hyg1F	ATAGGTCAGGCTCTCGCTGA	561	*Hpt* gene (T-DNA)
Hyg1R	ATCATACATGAGAATTAAGGG		
KanaF	AGACAATCGGCTGCTCTGAT	593	*NptII* gene (T-DNA)
KanaR	AGCCAACGCTATGTCCTGAT		
Hrm2F	TGCTTACAATAATATCCATCACCCA	94	HRM on *eIF4E1* region II
Hrm2R	AGGATCTTCCCACTTTGGCTC		
4er1F	GGTCCAAACAGTTCTTATAAATACCA	658	genomic *eIF4E1* Region I
4er1R	TTTTCCAATTTGAGCTTACGAA		
4er2F	CATGCACCTTGTTTGGTGAG	526	genomic *eIF4E1* Region II
4er2R	CCCGGACACTAACAACTGCT		
c4e1F	ATGGCAGCAGCTGAAATGGAGAGAACGATGTCG	696	*SleIF4E1* cDNA
c4e1R	CTATACGGTGTAACGATTCTTGGCATTTCTGTCG		
gapdhF	GGTTACAGTTCCCGTGTGATTGA	190 /195	*GAPDH* genomic/cDNA
gapdhR	TCAAAGATAACAACAGGGTTCCGTT		
